# Discovery and characterization of actionable tumor antigens

**DOI:** 10.1186/s13073-019-0642-x

**Published:** 2019-04-30

**Authors:** Grégory Ehx, Claude Perreault

**Affiliations:** 1 Institute for Research in Immunology and Cancer (IRIC), Université de Montréal, Montreal, Quebec H3T 1J4 Canada; 2 Department of Medicine, Université de Montréal, Montreal, Quebec H3T 1J4 Canada

## Abstract

The nature of the tumor antigens that are detectable by T cells remains unclear. In melanoma, T cells were shown to react against major histocompatibility complex (MHC)-associated peptides (MAPs) that are derived from exonic mutations. A recent multi-omic study of hepatocellular carcinomas suggests, however, that mutated exonic MAPs were exceedingly rare, bringing the accuracy of the current methods for antigen identification into question and demonstrating the importance of broadening tumor-antigen discovery efforts.

## Melanoma and immunotherapy: from solid breakthroughs to data misinterpretation

The current enthusiasm for cancer immunotherapy has been fueled in part by major breakthroughs in melanoma. Indeed, melanoma regression can now be achieved through the transfer of in vitro expanded tumor-infiltrating lymphocytes or through immune checkpoint therapy that targets regulatory pathways in T cells. These success stories are explained by two features of melanomas: i) they frequently form bulky and easily accessible tumors from which high numbers of tumor-infiltrating lymphocytes can be harvested, and ii) they are particularly immunogenic because they harbor a very high mutational load. As these approaches become established in the clinic, a key question remains as to the nature of the antigens that are capable of triggering therapeutic T-cell responses.

A series of seminal observations propelled interest in exon-derived mutated major histocompatibility complex (MHC)-associated peptides (mMAPs) as potentially crucial to T-cell responses to tumors. The presence of exonic mMAPs on melanoma tumors was demonstrated by mass spectrometry (MS) analyses, and these mMAPs were shown to elicit potent T-cell responses in functional assays. Like other MAPs, mMAPs are short peptides generated by intracellular digestion of proteins. After enzymatic trimming, these peptides bind to intracellular MHC molecules and are then exported at the cell surface. The cardinal feature of mMAPs is that they are coded by genomic regions that bear somatic cancer-specific mutations.

The excitement over the discovery of exonic mMAPs has, however, led to widespread acceptance of a speculative concept and to the introduction of a semantic bias. The unproven concept is that the repertoire of exonic mMAPs can be predicted (without MS validation) by combining exome sequencing and algorithms that predict MHC binding. The semantic reductionist bias was introduced when the term tumor-specific antigens (TSAs), also commonly referred to as neoantigens, was used to designate exonic mMAPs, implicitly suggesting that all TSAs were also exonic mMAPs. Formally, however, the terms TSA and neoantigen must encompass not only exonic mMAPs but rather all MAPs that are present only on cancer cells, irrespective of their genomic origin (exonic or not) and mutational status. This is not a trivial issue because exons represent only 2% of the genome, whereas 75% of the genome can be transcribed and potentially translated. Indeed, MS analyses identified MAPs derived from introns, 5′ UTRs, 3′ UTRs, long non-coding RNAs and intergenic regions [1]. Most of these non-exonic MAPs originate from short open reading frames of fewer than 100 codons [1]. A lower limit of 100 codons is arbitrarily used for gene prediction in genome annotation efforts, so the peptide-coding potential of short open reading frames remains underestimated.

## The MAP repertoire of cancer cells: insights from MS analyses of primary tumors

In this issue of *Genome Medicine*, Löffler and colleagues report on an important study of 16 primary human hepatocellular carcinomas. They performed exome and transcriptome sequencing and high-throughput shotgun MS analyses of the proteome and MAP repertoire, complemented by highly sensitive targeted MS analyses of selected MAPs [2]. Using the same pipeline, they identified a total of 12 exonic mMAPs in four melanomas [2]. The results obtained with hepatocellular carcinomas were striking. Using the same exome and transcriptome sequencing data, MHC-binding algorithms predicted that individual tumors would present an average of 118 exonic mMAPs. Remarkably, none of these predicted exonic mMAPs were detected by MS analyses. Two tentative conclusions can be drawn from these comprehensive analyses. First, consistent with recent reports [3], they cast serious doubts on the validity of predictions that are based solely on next-generation sequencing and MHC-binding algorithms. This is because current algorithms fail to take into account the numerous translational and posttranslational events that regulate MAP biogenesis and presentation [4]. Second, exonic mMAPs appear to be much less frequent in non-melanoma tumors such that, for most patients, they do not represent realistic therapeutic targets. The scarcity of exonic mMAPs in non-melanoma tumors is explained by their lower mutational load [2]. Over the past few months, similar findings were reported in a large cohort of chronic myelogenous leukemia patients [5], as well as for other non-melanoma tumor types [6].

How can we reconcile the scarcity of exonic mMAPs with the compelling evidence that many non-melanoma tumors display immunogenic MAPs? Arguably, the most parsimonious explanation is that the MAP repertoire of cancer cells contains a substantial amount of ‘dark matter’ (antigens that are not detected by the current approaches). In line with this, a recent study found that most TSAs present in human acute lymphoblastic leukemias and lung cancers derive from unmutated non-exonic sequences that are located in introns, intergenic regions and other noncanonical reading frames [6]. These aberrantly expressed TSAs (aeTSAs) were coded by RNAs that are not expressed in adult somatic cells, including medullary thymic epithelial cells (mTECs). mTECs deserve special attention here in view of their key role in establishing immune tolerance during the development of immature T cells (i.e., central tolerance), thereby limiting the potentially destructive responses of lymphocytes to host tissues, and because of their ability to promiscuously express more transcripts than other types of somatic cells [7]. All of the MAPs that are expressed in mTECs are expected to induce central immune tolerance and to be poorly immunogenic. In view of their cancer specificity, aeTSAs represent authentic TSAs or neoantigens. Their presence on cancer cells results from epigenetic changes that cause the expression of genomic sequences that are normally repressed in somatic cells. In particular, cancer-specific alterations in histone and DNA methylation commonly cause the overexpression of endogenous retroelements that can trigger both innate and adaptive immune responses [6, 8].

## Proposed guidelines for global analyses of the tumor-antigen landscape

The study by Löffler and colleagues shows that MS is the most robust method for high-throughput analyses of the MAP repertoire of tumor cells [2]. Notably, the breadth and sensitivity of MS analyses can be adjusted according to sample size and user preferences. We therefore suggest that MS analyses should be included at the discovery and/or validation stages of pipelines for tumor-antigen discovery. Furthermore, we strongly encourage the sharing of MS datasets via the SysteMHC Atlas [9]. In the short term, sharing of immunopeptidomic data will accelerate further analyses of the features of tumor antigens: whether they are shared among different tumors, their abundance in tumor cells (at the RNA and peptide levels), their immunogenicity and so on. In the long term, sharing will provide large validated tumor-antigen datasets that can be used as learning material for artificial neural networks, giving rise to more precise predictions.

Rapid progress in the field and the lack of a standardized nomenclature has led to some confusion in the classification of tumor antigens. We therefore offer a simple classification of tumor antigens based on three criteria: tissue-expression profile, genomic origin and mutational status (Table 1). Tumor-associated antigens (TAAs) are MAPs that show superior abundance on tumor cells but are nonetheless present on normal cells, and may therefore induce central immune tolerance [10]. TSAs are segregated into two main groups: mutated TSAs (mTSAs) and aeTSAs [6]. mTSAs derive from mutated DNA sequences that can be either exonic or non-exonic. aeTSAs result from the aberrant expression of transcripts that are not expressed in any normal somatic cell, including mTECs. Finally, a peculiar antigen family, the cancer-germline antigens (CGAs), sits astride the TAA and aeTSA categories.

**Table 1:** Classes of tumor antigens

	TAAs	mTSAs	aeTSAs
Genomic origin	Exonic ++++	Exonic++ Non-exonic ++	Exonic + (some CGAs) Non-exonic ++++
Expression in mTECs/normal tissues	Yes	No	No
Mutation	No	Yes	No
Shared among tumors	Often	No/rarely	Often
Derivation	Overexpression due to genetic and epigenetic changes	Somatic mutations	Epigenetic changes Aberrant splicing

The relative frequency of exonic vs non-exonic antigens is indicated using a scale of + (rare) to ++++ (very common) for each class of tumor antigens. aeTSA, Aberrantly expressed tumor-specific antigen; *CGA* Cancer-germline antigen, *mTEC* Medullary thymic cell, *mTSA* Mutated tumor-specific antigen, *TAA* Tumor-associated antigen

CGAs are coded by canonical exons that are normally expressed only by germ cells, and their aberrant expression in cancer cells is mostly driven by epigenetic alterations. However, some CGAs are expressed by adult mTECs [7]. Accordingly, we propose to classify those CGAs that are expressed in mTECs (or other somatic tissues) as TAAs, and those not expressed by any normal tissue (including mTECs) as genuine aeTSAs. One advantage of this simple classification is that antigen classes are linked to functional features. Thus, in contrast to TAAs, mTSAs and aeTSAs should not induce central immune tolerance and are expected to display superior immunogenicity. In addition, TAAs and aeTSAs may have two advantages as potential therapeutic targets over mTSAs: they are more numerous and evidence suggests that some are shared by many tumors [6, 10]. One major advantage presented by antigens that are shared by many tumors over mTSAs is they can be can be incorporated into off-the-shelf cancer vaccines. In principle, aeTSAs may offer the best of both worlds—being shared by many tumors without inducing central immune tolerance—but much more work is needed in order to evaluate the value of various classes of tumor antigens cogently.

A deep exploration of the immunopeptidomic dark matter will be necessary before conclusions can be reached. Substantial attention should be paid to three features of tumor antigens that will influence their potential for therapeutic translation: the proportion of tumors on which they are present, their abundance on tumor cells and their immunogenicity. Ultimately, clinical studies will determine what is the best way to target tumor antigens. The fact that multiple antigens can be incorporated into a single vaccine makes this approach very attractive. On the other hand, therapies that are based in T-cell receptors (using the injection of cells or bispecific biologics) would make it possible to launch potent attacks on tumor cells, even when the patient is immunodeficient.

## Abbreviations

aeTSA: Aberrantly expressed tumor-specific antigen;
CGA: Cancer-germline antigen
MAP: MHC-associated peptide
MHC: Major histocompatibility complex
MS: Mass spectrometry
mTEC: Medullary thymic epithelial cell
mTSA: Mutated TSA
TAA: Tumor-associated antigen
TSA: Tumor-specific antigen
